# Mapping of panda plumage color locus on the microsatellite linkage map of the Japanese quail

**DOI:** 10.1186/1471-2156-7-2

**Published:** 2006-01-12

**Authors:** Mitsuru Miwa, Miho Inoue-Murayama, Naoki Kobayashi, Boniface Baboreka Kayang, Makoto Mizutani, Hideaki Takahashi, Shin'ichi Ito

**Affiliations:** 1 Faculty of Applied Biological Sciences, Gifu University, Gifu 501-1193, Japan; 2 Department of Animal Science, College of Agriculture and Consumer Sciences, University of Ghana, Legon, Ghana; 3 Graduate School of Bioagricultural Sciences, Nagoya University, Nagoya 464-8601, Japan; 4 National Institute of Agrobiological Sciences, Tsukuba 305-8602, Japan

## Abstract

**Background:**

Panda (*s*) is an autosomal recessive mutation, which displays overall white plumage color with spots of wild-type plumage in the Japanese quail (*Coturnix japonica*). In a previous study, the *s *locus was included in the same linkage group as serum albumin (*Alb*) and vitamin-D binding protein (*GC*) which are mapped on chicken (*Gallus gallus*) chromosome 4 (GGA4). In this study, we mapped the *s *locus on the microsatellite linkage map of the Japanese quail by linkage analysis.

**Results:**

Segregation data on the *s *locus were obtained from three-generation families (*n *= 106). Two microsatellite markers derived from the Japanese quail chromosome 4 (CJA04) and three microsatellite markers derived from GGA4 were genotyped in the three-generation families. We mapped the *s *locus between *GUJ0026 *and *ABR0544 *on CJA04. By comparative mapping with chicken, this locus was mapped between 10.0 Mb and 14.5 Mb region on GGA4. In this region, the endothelin receptor B subtype 2 gene (*EDNRB2*), an avian-specific paralog of the mammalian endothelin receptor B gene (*EDNRB*), is located. Because *EDNRB *is responsible for aganglionic megacolon and spot coat color in mouse, rat and equine, *EDNRB2 *is suggested to be a candidate gene for the *s *locus.

**Conclusion:**

The *s *locus and the five microsatellite markers were mapped on CJA04 of the Japanese quail. *EDNRB2 *was suggested to be a candidate gene for the *s *locus.

## Background

**Figure 1:**
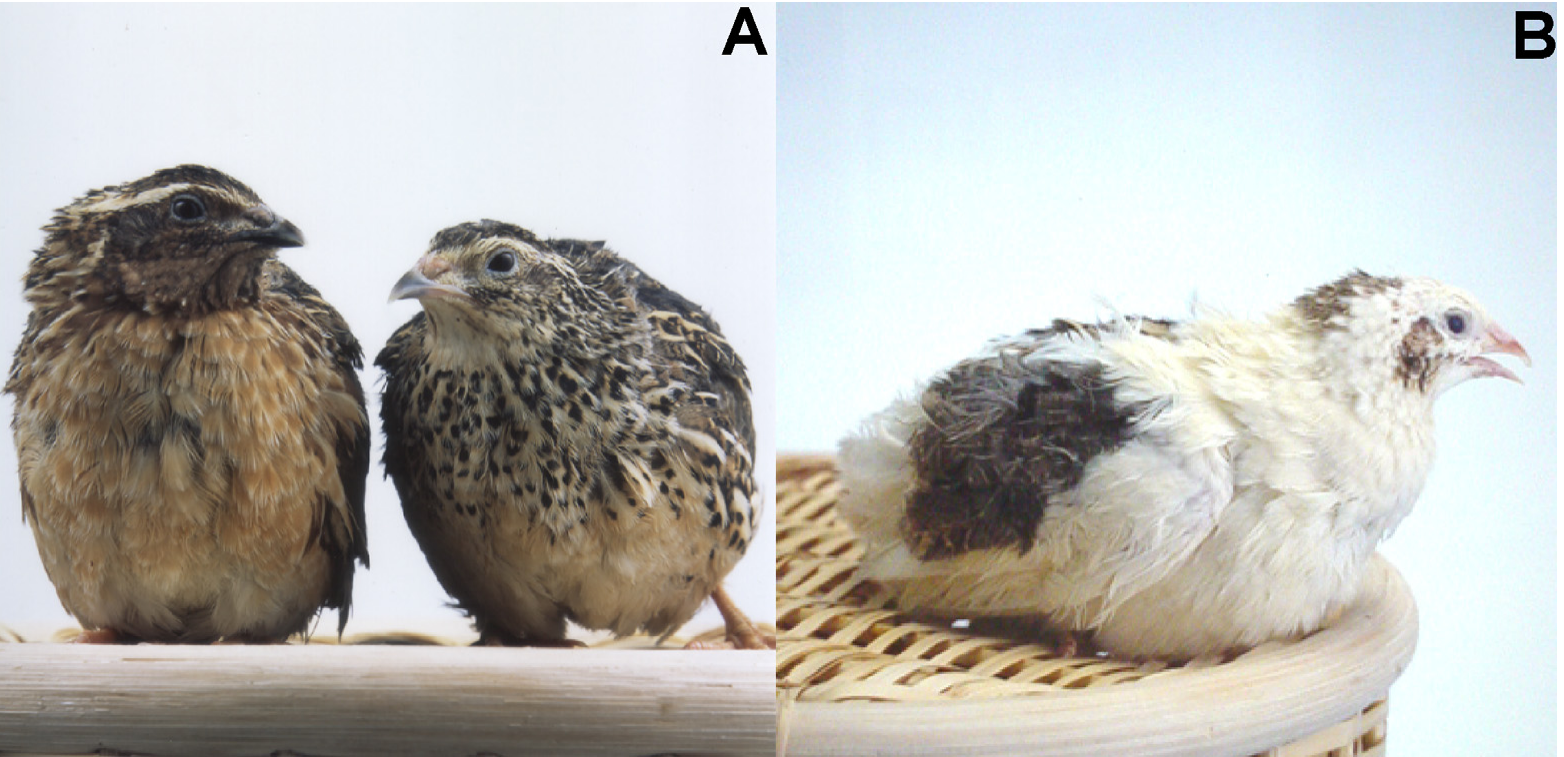
**The Japanese quail with wild-type (A) and panda (B) plumage color**. (A) A male (left) and a female (right) of the Japanese quail with the wild-type plumage. (B) The panda mutant shows overall white with spots of the wild-type plumage. There is no sex difference about the plumage pattern of the panda mutant.

Panda (*s*) is an autosomal recessive mutation, which causes overall white with spots of wild-type plumage in the Japanese quail (*Coturnix japonica*) (Figure 1) [1]. When they hatched, they have several brown spots in head, back and tail. After grew up, the spots become wild-type plumage color. Numbers and patterns of spots do not change in their life. A sex difference does not exist in panda mutant while adult wild-type male and female are distinguishable by their ventral plumage pattern. Recessive white (*wh*) [2] and dotted white (*s*^*dw*^) [3] mutants show phenotypes similar to panda, and *s*^*dw *^has been confirmed to be one of the multiple alleles at the *s *locus [4]. Although *s *and *s*^*dw *^mutants show similar phenotypes, they are distinguishable by the location and the size of spot. Because the Japanese quail is a suitable laboratory animal for research and a pilot animal for poultry [5,6], *s*, *s*^*dw *^and *wh *mutants have a potential for pigment cell research.

The *s *locus is included in the same linkage group as two classical markers, serum albumin (*Alb*) [7] and vitamin-D binding protein (*GC*) [8], in the Japanese quail. These markers are located on chicken chromosome 4 (GGA4) [9]. Because of high karyotype conservation and orthologous chromosomes between Japanese quail and chicken [10,11], the *s *locus is likely to be located on chromosome 4 of the Japanese quail (CJA04).

Recently, a microsatellite linkage map was constructed in the Japanese quail [12] and two plumage color loci, black at hatch (*Bh*) and yellow (*Y*), were mapped on this map [13]. This linkage map was also applied to map quantitative trait loci (QTLs) affecting commercial traits such as growth, feed consumption, egg production, tonic immobility and body temperature [14]. In the present study, to find the responsible gene for the *s *locus, we tried to map the *s *locus by the linkage analysis with microsatellite markers located on the candidate chromosome 4. Then, we searched for the candidate gene from the chicken draft genome sequence [15] corresponding to the region where *s *was mapped in the Japanese quail.

## Results and discussion

### Linkage analysis

**Figure 2:**
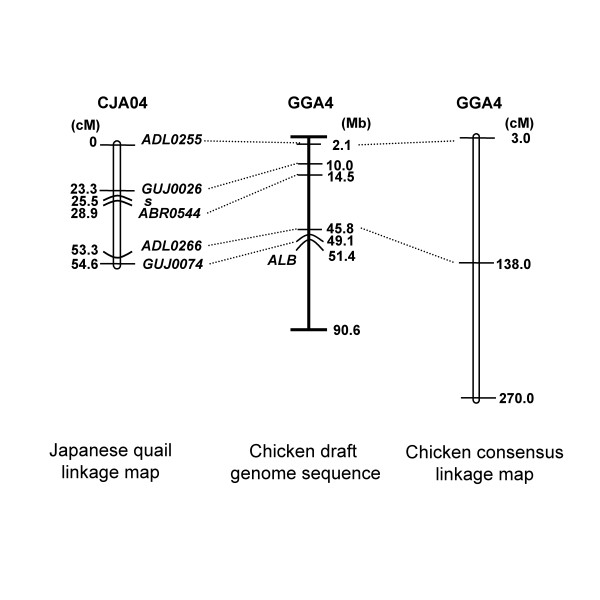
**The genetic linkage map of CJA04 and the corresponding position on GGA4**. Left: The sex averaged genetic linkage map of CJA04 based on a LOD Score threshold of 3.0 is shown with the estimated Kosambi map distances in cM. Middle: The positions of orthologous loci on the chicken draft genome sequence [9] [15] are shown with the physical map distances in Mb. Right: *ADL0255 *and *ADL0266 *were also located on the framework of chicken consensus linkage map with the estimated Kosambi map distances in cM [17]. Microsatellite markers named *GUJ *are original Japanese quail markers [26] [27] while those named *ADL *[17] or *ABR *[17] are original chicken markers.

The Q09 linkage group was suggested to be CJA04 because two microsatellite markers on the Q09 were mapped on GGA4 by BLAT search (Table 1) [16]. Thus, the Q09 linkage group was confirmed to be located on CJA04 in this study. Because there are only two microsatellite markers on CJA04, we tried to increase the available microsatellite markers from GGA4. Among thirty chicken microsatellite markers, eight were amplified in the Japanese quail and three were polymorphic in our resource family. Thus two original Japanese quail markers and three original chicken markers were selected for the linkage analysis [12,17,18] (Table 1). These markers were genotyped in the three-generation family for the linkage analysis, and the *s *locus was linked with *GUJ0026*, *ABR0544*, *ADL0266 *and *GUJ0074 *(LOD Score = 22.9, 21.1, 3.90 and 3.80, respectively). Although no linkage was indicated between *s *and *ADL0255*, *ADL0255 *was linked with *GUJ0026 *(LOD Score = 3.39). Thus, five microsatellite markers and the *s *locus were included in one linkage group and the estimated order of loci was *ADL0255-GUJ0026-s-ABR0544-ADL0266-GUJ0074 *(Figure 2). The number of markers located on the CJA04 was increased from two in the previous report [12] to six in the present study. The *s *locus is an easily distinguishable classical marker, and its assignment to the chromosome will further enhance the usefulness of the linkage map. The serum albumin locus (*Alb*) was located on GGA4 [9] and was classically linked with the *s *locus in the Japanese quail [7]. Because classical serum albumin locus (*Alb*) [7] was monomorphic in our three-generation family, it was not mapped on CJA04 in this study.

**Table 1:** Microsatellite markers selected for mapping the *s *locus.

		Japanese quail				Chicken		
			
				Nucleotide similarity between Japanese quail and chicken (%)				
							
Locus	Marker origin	GenBank accession number	T_A _ (°C)	5' flank	3' flank	GenBank accession number	References	Map position in the draft sequence of GGA4 (bp)
*ADL0255*	Chicken (GGA4)	AB038396	58	83 (71 nt)	100 (22 nt)	G01675	[17] [19]	2,088,640 – 2,089,022
*GUJ0026*	Quail (CJA04)	AB035836	60	91(87 nt)	90 (50 nt)	-	[26] [27]	10,067,404 – 10,067,587
*ABR0544*	Chicken (GGA4)	AB220928	55	89(108 nt)	93(107 nt)	AB186688	[18]	14,275,721 – 14,276,511
*ADL0266*	Chicken (GGA4)	AB220927	50	93 (15 nt)	89(25 nt)	G01686	[17] [18]	45,879,485 – 45,879,848
*GUJ0074*	Quail (CJA04)	AB063142	59	93 (253 nt)	83 (24 nt)	-	[27]	49,116,133 – 49,116,445

Nucleotide similarities of two original Japanese quail markers (*GUJ0026 *and *GUJ0074*) were calculated by BLAT search against the chicken draft genome sequence. Nucleotide similarities of three original chicken markers (*ADL0255*, *ABR0544 *and *ADL0266*) were calculated between the GenBank chicken sequence and quail sequence.

### Corresponding region on GGA4

Sequences of PCR products of *ABR0544 *and *ADL0266 *amplified from the Japanese quail genomic DNA were very similar to the corresponding chicken sequence indicating that they are orthologous loci (Table 1). *ADL0255 *was already confirmed to be a cross-species marker for the Japanese quail and chicken [19]. Thus, these three original chicken markers are useful for fine mapping of *s *in the Japanese quail. By BLAT search, all of the five microsatellite markers were mapped on GGA4 (Table 1) and the order of loci was the same between the Japanese quail and chicken (Figure 2). This result supports that of FISH indicating that CJA04 is homologous to GGA4 and the order of loci in this chromosome pair is conserved [10,11]. The distance between *ADL0255 *and *ADL0266 *in the Japanese quail linkage map was shorter than that in the chicken consensus linkage map [17]. This result suggests that recombinant frequency of CJA04 is smaller than that of GGA4 (Figure 2).

### Candidate gene

By comparative mapping with chicken, the *s *locus was suggested to be located between 10.0 Mb (*GUJ0026*) and 14.5 Mb (*ABR0544*) in an orthologous region of GGA4 (Figure 2). This region includes the endothelin receptor B subtype 2 gene (*EDNRB2*) as a candidate gene. *EDNRB2 *is strongly expressed in neural crest cells, melanoblasts, melanocytes, kidney and liver in the Japanese quail. *EDNRB2 *appears to be an avian-specific paralog of the mammalian endothelin receptor B (*EDNRB*) [20]. Alleles of *EDNRB *are responsible for aganglionic megacolon and spot coat color phenotype in mouse [21], rat [22,23] and equine [24]. Because avian *EDNRB2 *is not direct orthologue of the mammalian *EDNRB *[20], mammalian mutations such as aganglionic megacolon in mouse [21] are not exactly analogous to panda in the Japanese quail, even if the *s *locus does turn out to be in *EDNRB2*. The association study between *EDNRB2 *and panda mutation is underway by authors.

## Conclusion

The *s *locus and five microsatellite markers were included in the same linkage group which was designated as CJA04 in this study. From genome sequence information of the orthologous chicken GGA4, *EDNRB2 *was suggested to be a candidate gene for the *s *locus because of its function and chromosome location.

## Methods

### Three-generation family

A three-generation Japanese quail family was constructed to perform the linkage analysis. One wild-type (*+ /+*) male and one recessive homozygous (*s/s*) female were mated in the F_0 _generation to produce *+ /s *F_1 _progeny. Seven F_1 _birds were single-pair mated with seven *s/s *birds to produce 46 *+ /s *and 44 *s/s *birds in the F_2 _generation. Thus, a total of 106 birds were used as the resource family. Their plumage phenotypes were recognized when they hatched. This three-generation family was not included in any resource population which was used to construct the first-generation microsatellite linkage map of the Japanese quail [12]. DNA was extracted from peripheral blood using QIAamp DNA Blood Kit (Qiagen, Valencia, CA, USA).

### Microsatellite markers

PCR amplifications of five microsatellite markers (Table 1) were carried out on a PCR Thermal Cycler (TaKaRa Biomedicals, Shiga, Japan) in 10 μl reaction mixtures containing 14 ng of the DNA template, 0.3 μM of forward and reverse primers, 130 μM of dNTP, 10 mM Tris-HCl (pH 8.3), 50 mM KCl, 1.5 mM MgCl_2 _and 0.4 U AmpliTaq Gold (Perkin-Elmer, Foster City, CA, USA). After an initial incubation at 95°C for 9 min, amplification reactions were performed for 30 cycles each with denaturing at 95°C for 30 sec, annealing for 1 min at 50 to 60°C depending on the optimized annealing temperature of the primer used (Table 1), and extension at 72°C for 1 min. This was followed by a final cycle at 72°C for 5 min. PCR products were electrophoresed on an ABI Prism 3100 DNA Sequencer (Perkin-Elmer) and analysed using Genescan version 3.7 and the Genotyper version 3.7 softwares (Perkin-Elmer). To confirm whether the chicken primers of *ABR0544 *and *ADL0266 *amplified the orthologous Japanese quail microsatellite region, these PCR productswere cloned into TA cloning vector pCR2.1 (Invitrogen Corp., CA, USA) and sequenced by the dye termination method using ABI 3100 DNA Sequencer (Perkin-Elmer) (Table 1).

### Data analysis

Linkage analysis was performed based on a LOD Score threshold of 3.0 by CriMap version 2.4 software [25]. To search for the candidate gene, we examined the orthologous positions of the microsatellite markers mapped in the Japanese quail from the chicken draft genome sequence by BLAT search [16]. Because the GenBank sequence of *GUJ0026 *failed to give a BLAT match to the chicken draft genome sequence, we used longer sequence of *GUJ0026 *to detect the BLAT match.

## Authors' contributions

MM carried out molecular studies, constructed the Japanese quail family and drafted the manuscript. BBK and HT developed microsatellite markers. NK and MMZ constructed the Japanese quail family. MIM and SI coordinated the study and helped to draft the paper.
